# IMPACT-FH Study for Implementing Innovative Family Communication and Cascade Testing Strategies for Familial Hypercholesterolemia

**DOI:** 10.1016/j.jacadv.2024.101198

**Published:** 2024-08-14

**Authors:** Laney K. Jones, Gemme Campbell-Salome, Nicole L. Walters, Andrew Brangan, Kelly M. Morgan, Eric P. Tricou, Zoe T. Lindsey Mills, Mary P. McGowan, Samuel S. Gidding, Alicia M. Johns, H. Lester Kirchner, Alanna Kulchak Rahm, Amy C. Sturm

**Affiliations:** a Department of Genomic Health, Research Institute, Geisinger, Danville, Pennsylvania, USA; b Heart and Vascular Institute, Geisinger, Danville, Pennsylvania, USA; c Department of Population Health Sciences, Research Institute, Geisinger, Danville, Pennsylvania, USA; d Family Heart Foundation, Pasadena, California, USA; e Biostatistics Core, Research Institute, Geisinger, Danville, Pennsylvania, USA; f 23andMe, Sunnyvale, California, USA

**Keywords:** cascade testing, family communication, familial hypercholesterolemia, implementation strategies

## Abstract

**Background:**

Relatives of probands diagnosed with familial hypercholesterolemia (FH) should undergo cascade testing for FH.

**Objectives:**

The purpose of this study was to evaluate probands’ choices of innovative strategies to communicate their FH result with relatives and facilitate cascade testing uptake.

**Methods:**

Probands with an FH genetic result from the MyCode Community Health Initiative could choose to share their FH result with adult blood relatives via the Family and Healthcare Professional Packet (packet), family sharing and cascade chatbots (chatbot), and/or FH Outreach and Support Program (direct contact). Cascade testing uptake was measured as reported completion of genetic or cholesterol testing. Generalized estimating equations models were used to identify factors associated with testing.

**Results:**

One hundred seventy five probands received an FH result, median age was 58.9 (IQR: 44.9-69.3), and 58.9% were female. Probands shared information about 1,915 adult and 163 minor relatives (11.9 relatives per proband). Seventy percent of probands (121/175) selected at least one strategy for at least one adult relative. An average of 1.2 strategies was selected per adult relative. Cascade testing was completed for 26.6% (144/541) of adults with at least one strategy selected, 2.4% (33/1,374) of adults without a strategy selected, and 25.2% (41/163) of minor relatives. Factors associated with increased cascade testing uptake were selection of at least one strategy (6.32 higher odds), specifically, selection of direct contact (16.78 higher odds).

**Conclusions:**

Strategies implemented improved FH cascade testing uptake compared to previous estimates and in families where no strategy was selected. Overall uptake remains insufficient, which can be attributed to probands reluctance to select a strategy for many relatives.

Familial hypercholesterolemia (FH) is autosomal dominant and the most common cardiovascular genetic disorder, leading to premature atherosclerotic cardiovascular disease (ASCVD) and death, without early identification and treatment.^1^, ^2^, ^3^ Testing at-risk relatives of FH probands (‘cascade testing’) can be highly effective in identifying previously undiagnosed individuals with FH who require treatment.^4^, ^5^, ^6^ Cascade testing is cost-effective,^7^, ^8^, ^9^ reduces the average age at which FH is diagnosed,^10^ and prevents ASCVD and mortality.^1^

Geisinger’s MyCode Community Health Initiative (MyCode) and the MyCode Genomic Screening and Counseling (GSC) program^11^ has returned results to ∼4,450 patient-participants, including >560 with FH. The median age of FH patients in MyCode is 61 years, providing an opportunity for cascade testing of at least two generations of at-risk individuals in many families. Based on the data collected in 2018 for MyCode on 114 FH probands, there were 401 reported living first-degree relatives (FDRs) or 3.5 FDRs per FH proband. In that group (unpublished data), only 14 at-risk relatives from 6 FH families had completed cascade genetic testing (3.5%). These preliminary findings demonstrated the significant need for strategies to improve cascade testing uptake for FH families to benefit from the results of population screening for FH.

While there is support from payers, public health officials, and clinicians regarding the importance of cascade testing, how best to inform relatives of risk and implement cascade testing has yet to be determined.^12^ Novel implementation strategies for probands to communicate genetic risk with relatives have been developed but require further testing to assure generalizability.^13^ These strategies should provide education, offer support, and provide calls to action to assist probands and relatives in understanding their risk and empowering them to act.^14^ Despite success in other countries with innovative approaches to inform relatives of FH risk, these strategies have not been tested for effectiveness or acceptability in the U.S.^6^^,^^15^^,^^16^

We have previously reported on the development of implementation strategies for cascade testing informed by health communication theory and implementation science to improve cascade testing.^17^ These strategies include the following: 1) Family and Healthcare Professional Packet (packet); 2) Family Sharing Chatbot (FSC) and Cascade Chatbot (CC); and/or 3) FH Outreach and Support Program (direct contact).^17^ In this study, we offered these innovative strategies to probands with FH to evaluate proband choice for family communication with the aim of improving cascade testing uptake.

## Methods

### Study design

**Figure 1:**
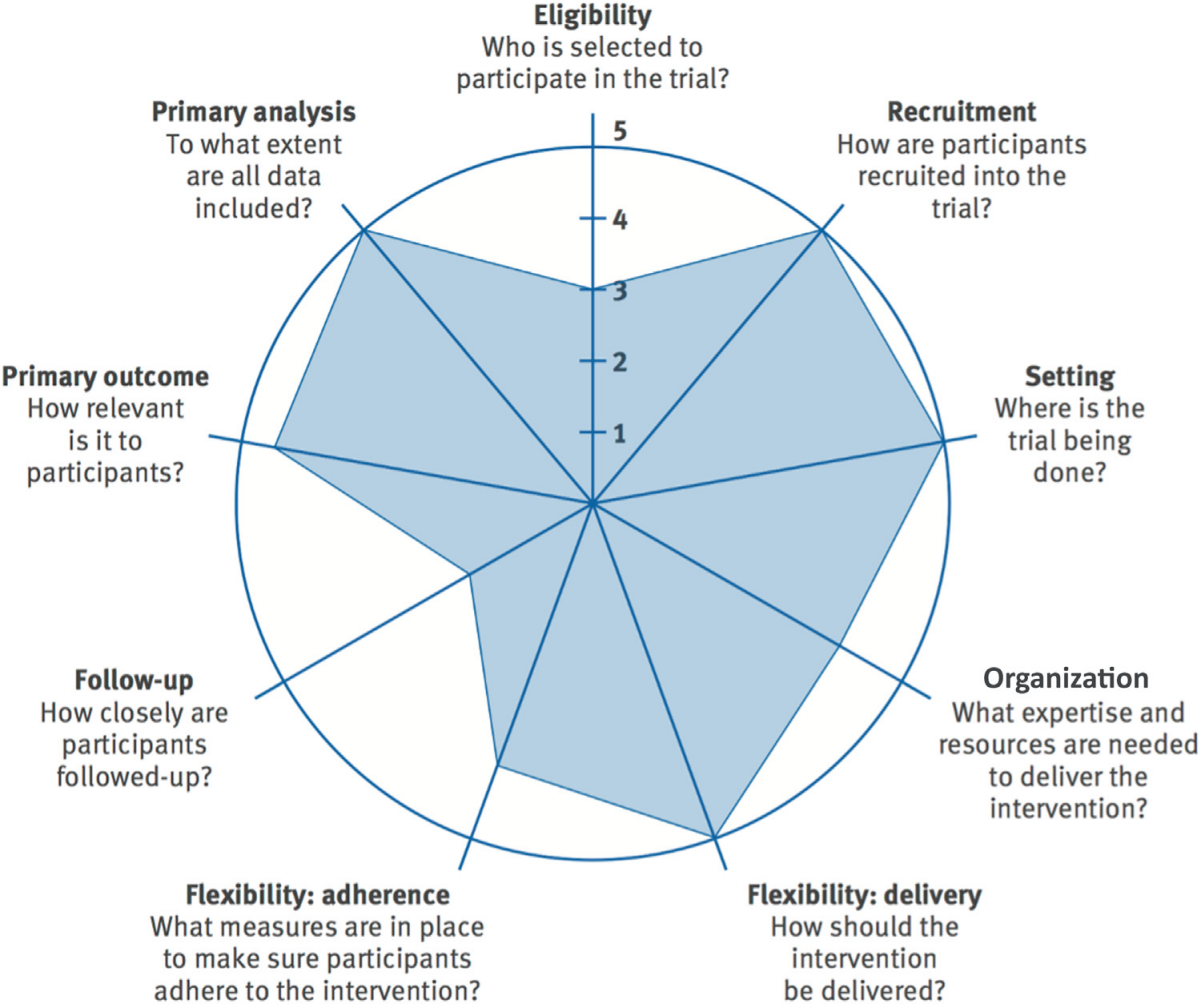
**PRagmatic-Explanatory Continuum Indicator Summary 2 Wheel for the IMPACT-FH Cascade Testing Program** The PRECIS-2 wheel depicts the ranking of each domain from least pragmatic (score of 1) to most pragmatic (score of 5).

Adapted from Loudon K et al. *BMJ*. 2015;350:bmj.h2147.

**Figure 2:**
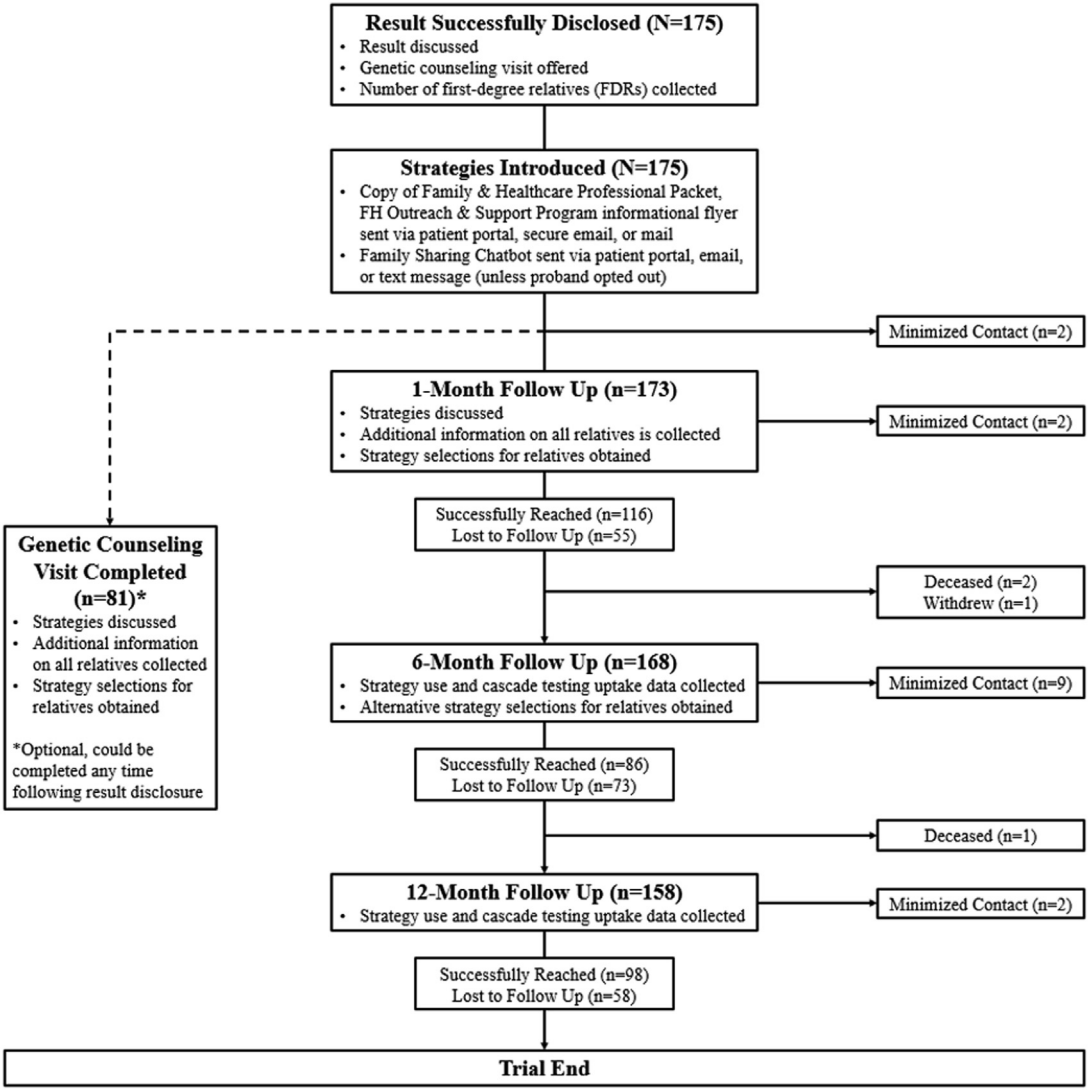
**Program Flow From FH Result Disclosure, to Strategy Selection, and to Study Follow-Up Visits** The trial flow depicts the probands follow-ups during the IMPACT-FH Cascade Testing Program.

A prospective pragmatic study utilizing implementation strategies to improve family communication and cascade testing for FH was conducted as part of the IMPACT-FH (‘Identification Methods, Patient Activation, and Cascade Testing for FH’) study (Central Illustration ).^18^ Figure 1 highlights the pragmatic design of the IMPACT-FH trial using the nine specified domains using the PRECIS-2 tool.^19^ These domains were scored by the study team. Higher scores reflect the extent to which the IMPACT-FH study was implemented in real-world practices. Further details on the scoring from very explanatory to very pragmatic of the nine domains and rationale are available in Supplemental Table 1. The trial was conducted at Geisinger from July 2021 to April 2023. An overview of the study timeline is shown in Figure 2. This study was approved by Geisinger’s Institutional Review Board (2020-0579).

**Central Illustration:**
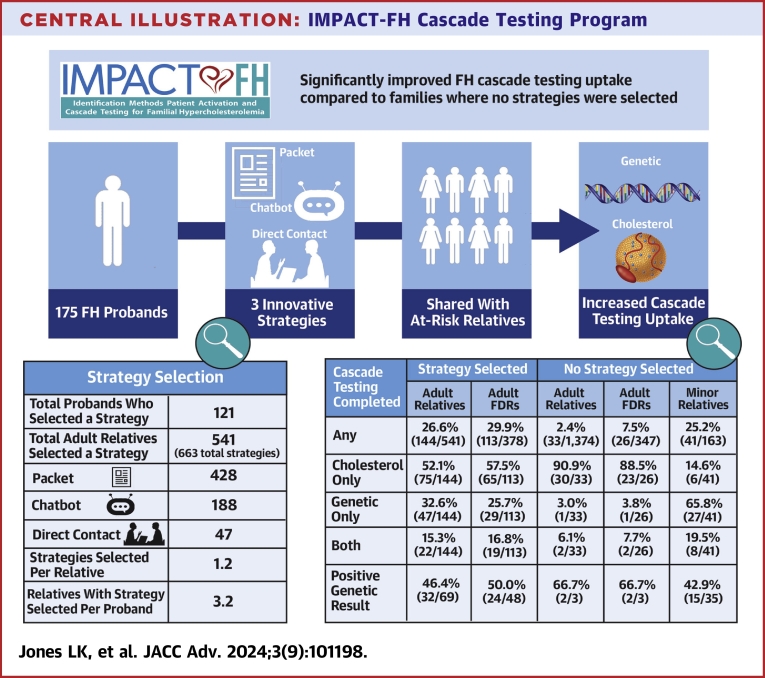
IMPACT-FH Cascade Testing Program

### Study setting

This study relies on previous genomic health efforts at Geisinger including MyCode and the MyCode GSC program.^20^^,^^21^ MyCode is a biobank of serum, blood, and DNA samples for health discovery research from patient-participants that have also undergone exome sequencing. MyCode launched in 2007 and began returning clinically actionable results to participants and their healthcare clinicians in 2015.^20^^,^^21^ When a pathogenic or likely pathogenic (P/LP) variant is identified in MyCode, the variant is confirmed in a CLIA-certified genetics laboratory prior to clinical report return.^22^ The standard clinical process is for the MyCode GSC program to return results to patient-participants (defined as the proband for this study) and their clinicians in the electronic health record.^11^ Upon return in the standard process, probands receive a packet that includes information about their result and a ‘Dear Family’ letter to share with at-risk relatives. They are also provided the option to receive a link to the FSC, a HIPAA-compliant web-based conversational tool that probands can utilize to send a separate CC link to their relatives. The CC provides relatives with information about the result in the family and contact information for the GSC team should they wish to schedule an appointment for cascade testing.

### Description of implementation strategies

Table 1 describes how the novel implementation strategies—the packet, FSC and CC, and direct contact—were offered to probands. Detailed descriptions on how these strategies were optimized, and co-developed, have been reported previously.^17^ Probands could choose different strategies for different relatives, and probands could choose different strategies at the different follow-up points.

**Table 1:** Optimized Implementation Strategies for the IMPACT-FH Cascade Testing Program

Name It	Family and Healthcare Professional (HCP) Packet		Family Sharing and Cascade Chatbot			FH Outreach and Support Program	
Define it	Physical or electronic document for relatives and their HCP about FH and cascade testing options		Web-based tool that probands can utilize to share information about FH and cascade testing options (including ordering a mail-in kit) with relatives			Direct contact to the relative and/or their HCP to discuss information about FH and cascade testing options with proband’s permission	
Specify it							
Actor	Study Team	Proband	Study Team	Proband	Study Team	Study Team	Genetic Counselor
Action	Sends the packet to the proband upon disclosure of FH result	Facilitates the communication of the proband’s FH result with their relatives and relative’s HCP	Sends the family sharing Chatbot to the proband upon disclosure of FH result	Utilizes family sharing Chatbot to send cascade Chatbot to relatives	Sends cascade Chatbot to relative(s)	Offers the program to probands for their relatives and/or relative’s HCP	Reaches out to relative and/or their HCP to discuss the proband’s FH result and cascade testing options
Action target	Proband	Relative	Proband	Relative	Relative	Proband	Relative and/or relative’s HCP
Dose	Minimum once and additional as requested by the proband	At the proband’s discretion how often and with which relatives	Minimum once and additional as requested by the proband	At the proband’s discretion how often and with which relatives	When requested by the relative	Minimum once and additional at completed touchpoints	Primer letter sent ahead of contact attempts if relative wishes to opt out. If relative does not opt out, up to 3 contact attempts as needed Up to 3 contact attempts as needed
Temporality (timepoint)	Upon disclosure of FH result and at subsequent touchpoints as requested	At the proband’s discretion of when and with which relatives	Upon disclosure of FH result and at subsequent touchpoints as requested	At the proband’s discretion of when and with which relatives	When requested by the relative	At the disclosure of FH result and at subsequent touchpoints	Primer letter sent approximately 2 weeks before first contact attempts When requested by proband
Implementation outcome	Penetration of the family and HCP packet to relative(s)	Uptake of at least one implementation strategy by a relative	Viewing of family sharing Chatbot by proband Penetration of cascade chatbot to relative(s)	Uptake of at least one implementation strategy by a relative	Uptake of at least one implementation strategy by a relative	Penetration of FH outreach and support program to relatives or their HCP	Uptake of at least one implementation strategy by a relative

FH = familial hypercholesterolemia; HCP = healthcare professional.

The optimized packet contains information about the proband’s FH result, including a personalized cover letter to the relative, describing the FH result and what it means for the relatives. It also includes detailed instructions on how to pursue both genetic and cholesterol-based cascade testing, a letter relatives can share with their own clinician, and the proband’s laboratory report containing the FH genetic result. The FSC is a proband-facing chatbot that explains why communicating the FH result to family is important and allows probands to share a CC link to at-risk relatives. The CC is a relative-facing chatbot that provides relatives with detailed information about FH and the importance of cascade testing and includes a genetic testing ordering module, allowing relatives to order a mail-in genetic testing kit for a fee ($20) within a 150-day window. This window is not specific to the chatbot or this study; it is the window set by the genetic testing laboratory for all “free” cascade testing available to relatives. If the packet or CC were chosen for a relative, sharing of these materials by the proband was voluntary and served as their HIPAA authorization.

Direct contact involves a genetic counselor with expertise in FH calling a relative and/or the relative’s clinician to discuss the FH result, importance of cascade testing, and navigating them through testing, if desired. The study staff coached the proband to give their relatives a “heads-up” about direct contact. The proband was asked if they wanted their relative(s) contacted directly by a genetic counselor, their relative’s clinician contacted by the study staff (so their clinician could forward this information to their relative), or both. If the genetic counselor was to contact the relative directly, a “primer” letter was also sent to the relative prior to the direct contact via mail, secure email, or the patient electronic health portal (if the relative also received care at Geisinger). This primer also allowed the relative to opt-out of further contact by the study team. In this situation, the study team recontacted the proband and offered the option to authorize the sharing of their FH genetic test result with their relative’s clinician instead. When direct contact was chosen, genetic counselors were given authorization by the proband to share their information with their relatives or their relative’s clinicians via a verbal HIPAA authorization.

### Proband eligibility

Probands had to be 18 years or older with a clinically confirmed P/LP genetic variant for FH in either *LDLR*, *APOB*, or *PCSK9*, which was returned to them by a genetic professional from July 1, 2021 to April 1, 2022 through the MyCode GSC program. FH results were disclosed per MyCode’s standard clinical process to inform participants of their FH risk variant, described above.^11^ To be included, probands had to have a preferred language of English, provide information on at least one living at-risk blood relative, and be introduced to the strategies. Probands were excluded if they received multiple genetic results or had prior clinical genetic testing for FH.

### Proband notification and study procedures

For the IMPACT-FH study, during the MyCode FH result disclosure or a subsequent genetic counseling appointment, probands were introduced to the three strategies and could select the packet, CC, or direct contact program to communicate about the FH result to their relatives. Study genetic counselors collected names, date of birth/age, and degree of relationship for each of the proband’s living blood relatives with whom they wanted to share their FH result with. In alignment with the MyCode GSC protocol, if a proband had been lost to follow-up for result return, a separate 1-month follow-up call would be attempted to return the FH result to the patient, include them in the trial, and collect strategy selections. If successfully returned at that 1-month follow-up call, the patient would follow the study protocol and follow-up schedule.

### Study follow-ups

Probands were contacted by the study team at 1-, 6-, and 12-month post return of the FH result (Figure 2). The 1-month follow-up contact offered an additional opportunity to begin or expand upon the proband’s strategy choices for each adult relative, the relative's information, and offered additional packets or to re-send the FSC/other materials as needed/requested. At the 6-month follow-up, the study team collected proband-reported outcomes on family communication choices (which strategies were sent by the proband) and cascade testing uptake. The proband was encouraged to select an alternative strategy for adult relatives that remained untested. For example, if the proband chose to use direct contact for an adult relative and testing was not performed by 6 months, then the proband would be offered the FSC/CC or packet for that relative at 6 months, and cascade testing uptake would be measured at 12 months. At the 12-month follow-up and final touchpoint, no additional strategies could be selected, and the study team collected proband-reported outcomes on family communication, strategy use, and cascade testing uptake.

### Outcomes

The study team documented all at-risk relatives identified by probands. Adult relatives were defined as all living blood relatives. FDRs were defined as relatives who were parents, full siblings, and adult children (age >18 years old). Minors were any relative less than the age of 18 years old. Family communication choice was defined as the selection of a strategy (packet, CC, or direct contact program) by the proband to share their result with their relatives and recording of the strategy or strategies selected. Cascade testing was defined as relatives completing either cholesterol and/or genetic cascade testing. Cholesterol testing was reported to the study team at one of the study follow-ups or reported by the proband or relative during an interview with the study team. For Pennsylvania resident relatives, options for cascade testing included scheduling an appointment with a genetic counselor at Geisinger, ordering through the CC, or by being directly contacted by a genetic counselor from the study team via the direct contact program. For out-of-state relatives, options for cascade testing included scheduling an appointment with a genetic counselor or clinician near them or ordering through the CC. For cascade testing not facilitated through Geisinger, the relative needed to provide consent/authorization for their data to be released from the clinical genetic testing laboratory to Geisinger. We examined uptake of cascade testing at 12 months by adult relatives who had a strategy selected for them by the proband. We also assessed the number of relatives that did not have a strategy selected for them by their proband but completed cascade testing for FH. This population includes all minors for whom the strategies were not intended. We assessed the factors associated with the uptake of cascade testing of cholesterol, genetic, or both types of testing, including proband demographics, proband completing a genetic counseling appointment, and proband selection of type and one or more strategies for adult relatives.

### Statistical analysis

All statistical analyses were conducted in SAS Enterprise Guide Version 8.3 (SAS Institute Inc). Data for probands, adult relatives, and minor relatives were described using medians and interquartile ranges (IQR) for continuous variables, and frequency and percentage for categorical variables. Comparisons between any strategy selected and no strategies selected, and between those that completed and did not complete cascade testing at 12 months, were performed using Generalized estimating equations (GEE) due to the inherent family clustering. The independence correlation structure was used to account for the clustering. For the binary outcomes, the logistic regression GEE model was specifically used. To identify factors associated with cascade screening uptake, the results from the individual models were combined to estimate adjusted effects. This model included indicator variables for the 3 implementation strategies selected and demographic variables. Demographic variables included age and sex of the relatives. The analysis was repeated collapsing the three strategies as any selected and comparing against none selected. Results of all GEE models are reported as odds ratios and 95% CIs. Lastly, the individual cascade screening uptake (ie, genetic, cholesterol) were analyzed separately.

## Results

### Proband and relative demographics

A total of 175 probands received a genetic result for FH from MyCode and were eligible to be included in the IMPACT-FH study and cascade testing program (Figure 2). The median age of probands was 58.9 years (IQR: 44.9-69.3 years) and 58.9% (103/175) were female (Table 2). There were 8 families where 2 MyCode participants received an FH result and 1 family where 3 individuals received an FH result from MyCode; all were considered probands. A total of 46.2% (81/175) of the probands completed an appointment with a genetic counselor, 67.0% (116/173) completed the 1-month follow-up, 51.2% (86/168) completed the 6-month follow-up, and 62.0% (98/158) completed the 12-month follow-up (Figure 2).

**Table 2:** Demographics of Probands and Relatives^a^

	Probands (n = 175)	Relatives		
		All (N = 2,078)	Adults (n = 1,915)	Minors (n = 163)
Age at FH result disclosure, y	58.9 (44.9-69.3)	48.0 (27.0-64.0)	53.0 (36.0-65.5)	8.1 (4.2-14.3)
Missing	0	1,022	1,007	15
Female	103 (58.9%)	938 (49.7%)	863 (49.8%)	75 (48.1%)
Missing	0	189	182	7
Race				
White	170 (97.1%)	128 (96.2%)	105 (99.1%)	23 (85.2%)
Other^a^	5 (2.9%)	5 (3.8%)	1 (0.9%)	4 (14.8%)
Missing	0	1,945	1,809	136
Ethnicity				
Non-Hispanic or Latino	172 (98.3%)	113 (100.0%)	87 (100.0%)	26 (100.0%)
Other^b^	3 (1.7%)	0 (0.0%)	0 (0.0%)	0 (0.0%)
Missing	0	1,965	1,828	137
State				
PA	170 (97.1%)	638 (88.2%)	536 (87.3%)	102 (93.6%)
Not PA	5 (2.9%)	85 (11.8%)	78 (12.7%)	7 (6.4%)
Missing	0	1,355	1,301	54
Insurance type				
No insurance	5 (2.9%)	0 (0.0%)	0 (0.0%)	0 (0.0%)
Private insurance only	77 (44.0%)	50 (74.6%)	37 (71.2%)	13 (86.7%)
Medicaid only	18 (10.3%)	4 (6.0%)	2 (3.8%)	2 (13.3%)
Medicare only	8 (4.6%)	3 (4.5%)	3 (5.8%)	0 (0.0%)
Private insurance and medicaid	4 (2.3%)	0 (0.0%)	0 (0.0%)	0 (0.0%)
Private insurance and medicare	57 (32.6%)	8 (11.9%)	8 (15.4%)	0 (0.0%)
Medicaid and medicare	3 (1.7%)	1 (1.5%)	1 (1.9%)	0 (0.0%)
Medicare and tricare/military	2 (1.1%)	0 (0.0%)	0 (0.0%)	0 (0.0%)
Private insurance, Medicaid, and Medicare	1 (0.6%)	0 (0.0%)	0 (0.0%)	0 (0.0%)
Prefer not to answer	0 (0.0%)	1 (1.5%)	1 (1.9%)	0 (0.0%)
Missing		2,011	1,863	148
Variant 1 gene				
APOB	63 (36.0%)	9 (79.1%)	7 (21.9%)	2 (18.2%)
LDLR	112 (64.0%)	34 (79.1%)	25 (78.1%)	9 (81.8%)
Missing		2,035	1,883	152
Completed a genetic counseling appointment	81 (46.3%)			

Values are median (IQR), n (%), or n.

a Includes Asian, Black or African American or prefer not to answer.

b Includes Hispanic or Latino or prefer not to answer.

At study follow-ups, probands reported on strategies sent to relatives and uptake of cascade testing. Probands shared information on a total of 2,078 relatives (11.87 relatives per proband). Of the 2,078 relatives with demographic information available, they had a median age of 48.0 years (IQR: 27.0-64.0 years; *P* < 0.0001 vs probands), half female, and mostly white (96.2%). There were 1,915 adults (725 were FDRs) and 163 minors (68 were FDRs) (Table 2). There was an average of 10.94 ± SD 8.98 adult relatives and 4.19 ± 2.10 adult FDRs reported per proband.

### Family communication strategy

Of the 175 probands, 121 (69.1%) selected at least one strategy for at least one adult relative with a total of 663 strategies selected for 541 relatives; an average of 1.2 strategies was selected per relative. Packets were selected for 428, CCs for 188, and direct contact for 47 relatives (Supplemental Table 2). An average of 3.2 relatives per proband had a strategy selected. Most often, the proband selected the strategy for the relative at one of the study follow-ups. No strategy was selected in 1,374/1,915 (71.7%) relatives. The most common strategies selected for adult relatives were packet only (58.8%, 318/541), packet and CC (16.8%, 91/541), and CC only 15.7% (85/541). The most common strategies sent for adult FDRs were packet only (61.6%, 233/378), packet and CC (17.5%, 66/378), and CC only 12.2% (46/378). Probands were more likely to select a strategy for their relative if they completed a genetic counseling appointment (OR: for packet 1.69 (95% CI: 1.07-2.65), *P* = 0.0236; for CC 2.17 (95% CI: 1.4-3.4), *P* = 0.0008; and for direct contact 2.74 (95% CI:1.7-4.4), *P* < 0.0001 versus none selected). Older age significantly impacted strategy selected by proband (*P* = 0.01). Selection of the packet and direct contact significantly differed in relative’s age from the no strategy selected (*P* = 0.002 and *P* = 0.05, respectively).

### Cascade testing uptake

**Figure 3:**
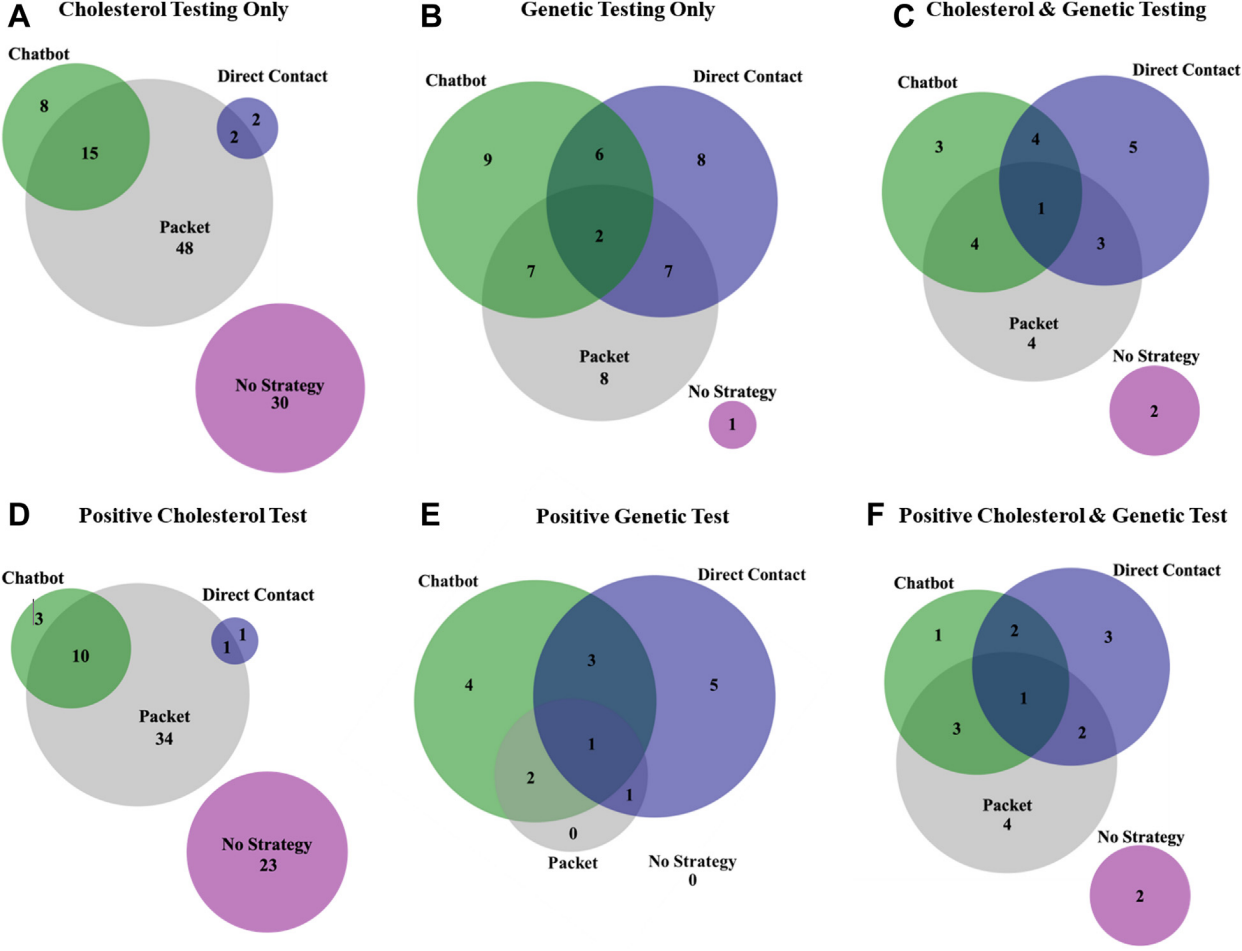
**Venn Diagram Showing Methods and Outcomes of Cascade Testing Uptake by Communication Strategy Utilized by the Relative** The Venn diagram shows the method (A, cholesterol testing; B, genetic testing; C, cholesterol and genetic testing) and result (D, positive cholesterol test; E, positive genetic test and negative or missing cholesterol test for FH; F, positive cholesterol and genetic test result) of cascade testing by the relative in relation to the implementation strategy utilized: Family and HCP Packet—packet (gray), Cascade Chatbot (green), and FH Outreach and Support Program—direct contact (*purple*).

Figure 3describes the selected strategies for adult relatives and result of cascade testing including positive result from cholesterol testing, genetic testing, and cholesterol and genetic testing.

#### Strategy selected

Cascade testing was completed by at least one relative in 44.6% (52/121) of probands who selected a strategy to share their FH result. Cascade testing was completed in 26.6% (144/541) of adult relatives (29.9% [113/378] of adult FDRs) that had a strategy selected for them (Supplemental Table 3). Of the 144 adult relatives that completed testing, 52.1% (75/144) completed cholesterol testing only, 32.6% (47/144) completed genetic testing only, and 15.3% (22/144) completed both. There were similar rates of cascade testing in adult FDRs: 57.5% (65/113) completed cholesterol testing only, 25.7% (29/113) completed genetic testing only, and 16.8% (19/113) completed both. Genetic cascade testing was completed in 42.5% (48/113) of FDRs compared to 3.5% in MyCode FDRs prior to implementation of the strategies (*P* < 0.0001). Of the 69 relatives that had genetic testing, 46.4% (32/69) had a positive result; of the 48 FDRs that completed genetic testing, 50.0% (24/48) had a positive result.

#### No strategy selected

Cascade testing was completed in 2.4% (33/1,374) of adult relatives (7.5% [26/347] FDRs) that did not have a strategy sent by a proband (Supplemental Table 3). Of the 33 adult relatives that completed testing, 90.9% (30/33) completed cholesterol testing only, 3.0% (1/33) completed genetic testing only, and 6.1% (2/33) completed both. There were similar rates in FDRs: 88.5% (23/26) completed cholesterol testing only, 3.8% (1/26) completed genetic testing only, and 7.7% (2/26) completed both. Of the 3 relatives that completed genetic testing, all 3 were FDRs and 66.7% (2/3) had a positive FH result.

A quarter of minors (41/163) completed cascade testing. Of the 41 minors that completed cascade testing, 14.6% (6/41) completed cholesterol testing only, 65.8% (27/41) completed genetic testing only, and 19.5% (8/41) completed both. Of the 35 minors that completed genetic testing, 42.9% (15/35) had a positive FH result.

### Factors associated with cascade testing uptake

When any strategy was selected for adult relatives, the odds of completing cascade testing were 6.32 times higher (95% CI: 3.17-12.61) than those without a strategy selected (Table 3) after adjusting for relative’s age and sex. There was no statistically significant difference across strategies selected by probands when only cholesterol testing was completed for adult relatives or FDRs. There were significant differences across strategies selected for adult relatives and FDRs when only genetic testing was completed and when both cholesterol testing and genetic testing were completed (*P* < 0.0001 and *P* < 0.0001, respectively). For instance, for relatives that had a packet selected for them, 4.8% completed only genetic testing; whereas for CC and direct contact, 13.8% and 39.4% of relatives completed only genetic testing, respectively. When direct contact was selected for adult relatives, the odds of completing cascade testing were 16.78 times higher (95% CI: 8.53, 15.51) than no strategy selected (Table 3). There was a statistically significant difference in cascade testing uptake across strategies selected for adult relatives. Relatives that had packet, CC, and direct contact had 23.6%, 30.3%, and 80.8% cascade uptake completed, respectively (*P* < 0.0001). Results for adult FDRs were similar: packet, CC, and direct contact had 25.6%, 38.2%, and 75.8% cascade testing uptake completed, respectively (*P* < 0.0001). No statistically significant differences between completion of cascade testing uptake and other demographic characteristics for probands, adult relatives, or adult FDRs were found.

**Table 3:** Adjusted Odds Ratio of Cascade Testing Uptake for Each Communication Strategy Selected

	Family and HCP Packet vs No Strategy Selected	Cascade Chatbot vs No Strategy Selected	FH Outreach and Support Program vs No Strategy Selected	Any Strategy vs No Strategy Selected
Cascade testing uptake				
Relatives	5.48 (2.70-11.12), *P* < 0.0001	7.50 (3.63-15.51), *P* < 0.0001	16.78 (8.53-15.51), *P* < 0.0001	6.32 (3.17-12.61), *P* < 0.0001
FDRs	2.50 (1.24-5.01), *P* = 0.0099	3.69 (1.79-7.60), *P* = 0.0004	7.18 (3.72-13.88), *P* < 0.0001	2.82 (1.43-5.58), *P* = 0.0028
Cholesterol testing only completed				
Relatives	4.02 (1.96-8.22), *P* = 0.0001	3.61 (1.62-8.06), *P* = 0.002	2.04 (0.52-7.93), *P* = 0.3052	3.80 (1.88-7.71), *P* = 0.0002
FDRs	2.01 (0.98-4.11), *P* = 0.06	1.83 (0.47-7.15), *P* = 0.14	1.00 (0.23-4.30), *P* = 0.9992	1.97 (0.97-3.98), *P* = 0.0590
Genetic testing only completed				
Relatives	30.59 (3.90-239.95), *P* = 0.001	68.18 (9.41-494.25), *P* < 0.0001	231.31 (31.62-1,692.05), *P* < 0.0001	46.78 (6.43-340.33), *P* = 0.0001
FDRs	---	---	---	13.84 (1.85-103.25), *P* = 0.0104
Any Genetic testing completed				
Relatives	---	---	---	23.05 (5.07-104.81), *P* < 0.0001
FDRs	---	---	---	7.84 (1.69-36.37), *P* = 0.0086
Any cholesterol testing only completed				
Relatives	4.33 (2.08-9.00), *P* < 0.0001	4.62 (2.04-10.46), *P* = 0.0002	7.00 (2.89-16.99), *P* < 0.0001	4.48 (2.18-9.24), *P* < 0.0001
FDRs	2.13 (1.02-4.44), *P* = 0.04	2.46 (1.07-5.63), *P* = 0.03	3.69 (1.56-8.70), *P* = 0.003	2.27 (1.10, 4.68), *P* = 0.0266

Values are OR (95% CI). Results are adjusted for relative’s age and sex.

FDR = first degree relative; FH = familial hypercholesterolemia; HCP =Healthcare Professional.

## Discussion

The underdiagnosis of FH is a significant public health problem.^23^ Cascade testing is a proven, evidence-based, cost-effective method to diagnose FH at younger ages and prevent ASCVD.^6^, ^7^, ^8^, ^9^, ^10^ The IMPACT-FH study was unique due to its pragmatic design and allowing the proband multiple options to facilitate sharing their FH results with relatives which, to our knowledge, has not been previously tested. We found that 69% of probands selected at least one strategy to share their FH result with their adult relatives. Almost one-third of adult relatives that had a strategy selected completed cascade testing (32.6%). Factors that influenced cascade testing uptake included type of cascade test, selection of the CC and direct contact strategies, and completion of a genetic counseling appointment by the proband. The selection of at least one strategy resulted in higher odds (6.32 times) of completing cascade testing. These findings demonstrate the importance of probands choosing a strategy for relatives to improve cascade testing and highlight persistent barriers of family communication on cascade testing uptake.

Offering a family letter is common practice during return of genetic results to patients to facilitate family sharing and may feel like a comfortable option for clinicians and families. Our optimized packet was the most frequently selected strategy; it resulted in the lowest uptake of cascade testing when compared to the other available strategies. Previous research has similarly found that passive strategies like a family letter have yielded suboptimal FH cascade testing uptake.^24^ Results on uptake of the chatbots and direct contact by probands further demonstrates that innovative, direct approaches are acceptable and feasible options^25^ and builds on previous research, suggesting these strategies can improve FH identification.^6^^,^^26^^,^^27^ Further, direct contact was one of the most effective strategies for improving cascade testing uptake; however, it was chosen less often than the packet or CC.

Cascade testing results have been variable across programs in the U.S. and abroad.^6^ In the IMPACT-FH cascade testing program, the uptake of cascade testing was significantly lower in those relatives for whom a strategy was not selected by their proband. We found that strategies were more likely to be selected by probands that completed a visit with a genetic counselor. Miller et al. found in their cascade testing trial that probands with a genetic result for FH had higher rates of cascade testing uptake (43.9% vs 21.4%) than those without a genetic resul.^27^ Our study supports that when the proband has a genetic result, we saw similar rates of cascade testing uptake; however, we are unable to confirm if having a genetic test result for FH increases rates of cascade testing. Ajufo et al. found that only 12% of probands communicated their FH result with a relative which led to those relatives being cascade tested.^28^ This further emphasizes that probands’ choice of who, why, and when they share their FH result with relatives is a rate-limiting step towards getting relatives tested for FH. Further research needs to address how to activate probands to share their result with relatives, in general, and for the development of additional strategies to help ease the burden of sharing FH risk information with relatives.

### Study Limitations

We likely have underestimated the rates of cascade testing in relatives because we relied on the probands to report demographics and cascade testing uptake by their relatives. Our study did allow for relatives to consent for the genetic testing laboratory to share results back with our study team, which we used as an objective measure of cascade testing; however, some may not have consented, potentially due to privacy concerns, which may have caused further underestimation. The study population was majority White and non-Hispanic, which may limit generalizability of the strategies for cascade testing uptake to more racially or ethnically diverse patient populations. Our study does demonstrate a significant improvement in cascade testing in a population that is rural and medically underserved, suggesting this study may have begun crucial work in overcoming barriers to cascade testing. Future research should seek to further optimize the strategies to other patient populations and replicate the pragmatic trial to improve FH identification in other healthcare settings. More research is needed on the implementation, effectiveness, and scalability of these strategies to facilitate their dissemination to address the public health problem of FH under identification.

## Conclusions

The innovative and optimized strategies implemented in the IMPACT-FH cascade testing program significantly improved FH cascade testing uptake compared to previous rates in a population genomic testing program and compared to families where no strategies were selected. We found direct contact was the most effective strategy for improving cascade testing uptake; it was also the least frequently chosen by probands. Future research should also examine the cost and feasibility of the optimized strategies to scale effective methods to other healthcare systems and settings.

PERSPECTIVES **COMPETENCY IN INTERPERSONAL & COMMUNICATION SKILLS:** It is important to offer choice of strategies to probands to facilitate communication of FH results with relatives.

**TRANSLATIONAL OUTLOOK 1:** When the FH Outreach and Support Program was selected, it had the highest odds of relatives undergoing cascade testing but how this program can be scaled and its cost require further investigation.

**TRANSLATIONAL OUTLOOKS 2:** Cascade testing programs should be personalized for every family and there is not a one-size-fits all approach and additional strategies should be investigated to help facilitate this conversation.

## Funding support and author disclosures

Dr Jones is a consultant for Novartis. Dr McGowan is on the advisory board for Novartis. Dr Gidding is a consultant for Esperion. Dr Sturm is an employee and stockholder in 23andMe; and on the advisory board for Nest Genomics. Research reported in this publication was supported by the 10.13039/100000050National Heart, Lung, And Blood Institute of the 10.13039/100000002National Institutes of Health under Award Number R01HL148246. The content is solely the responsibility of the authors and does not necessarily represent the official views of the National Institutes of Health. All other authors have reported that they have no relationships relevant to the contents of this paper to disclose.
