# Supplementary material for: IMPACT-FH Study for Implementing Innovative Family Communication and Cascade Testing Strategies for Familial Hypercholesterolemia
Source: JACC Adv. 2024 Aug 14;3(9):101198. doi: 10.1016/j.jacadv.2024.101198 (PMC11375316; doi:10.1016/j.jacadv.2024.101198)
Supplement: Supplementary Tables [file mmc1.docx]

**Supplemental Table 1.** PRECIS-2 Domains, Definitions, Score, and Rationale for Pragmatic Trial

| **Domain** | **Definition** | **Score** | **Rationale** |
| --- | --- | --- | --- |
| **Eligibility** | To what extent are the participants in this study are like those who would receive these strategies if it was part the MyCode GSC program? | 3 | At the beginning of the study, the score for eligibility was higher (4.5) because the inclusion criteria included that the disclosure the FH result directly to the proband (could not be lost to follow-up). Once the trial began, additional criteria were added which lowered the score to 3 including:  inclusion: 1) proband had to provide information about 1 at-risk relative and be introduced to strategies, and 2) out-of-state probands who are no longer receive care at Geisinger if they meet the other inclusion criteria and  exclusion: 1) non-English speaking, 2) more than 1 genomic risk variant at the same time, 3) disclosure via patient portal and not reached by phone, 4) inadequate amount of sample to send to Invitae, 5) variant reclassification from variants of unknown significance to likely pathogenic at Invitae, and 6) clinically identified previously but result was missed by pipeline |
| **Recruitment** | How much extra effort is made to recruit participants over and above what that would be used in the MyCode GSC program? | 5 | This study followed the same process as the MyCode GSC program for result disclosure and recruitment. |
| **Setting** | How different is the setting of the this study and the MyCode GSC program? | 5 | There is no difference in setting for this study. |
| **Organization** | How different are the resources, provider expertise and the organization of care delivery in this study and those available in the MyCode GSC Program? | 4 | Participants in this study will receive optimization strategies to communicate FH results with their relatives. Provider expertise ^14^is similar to the process in the MyCode GSC program. Organization of care delivery is the same. |
| **Flexibility (delivery)** | How different is the flexibility in how the strategies are delivered and the flexibility likely in the MyCode GSC program? | 5 | This study had similar flexibility to MyCode GSC program. |
| **Flexibility (adherence)** | How different is the flexibility in how participants must adhere to the strategies and the flexibility likely in the MyCode GSC program? | 4 | This study provided encouragement to participate in the strategies provided and had more frequent touchpoints with participants than MyCode GSC program. |
| **Follow-up** | How different is the intensity of measurement and follow-up of participants in this study and the likely follow-up in the MyCode GSC program? | 2 | This study had more intensive follow-up including 1-, 6-, and 12-month follow-up touchpoints than the GSC program. RA offering modalities, unscheduled visits, multiple follow-up points to collect additional data (outside of GSC program) |
| **Primary outcome** | To what extent is the trial's primary outcome relevant to participants? | 4.5 | Outcomes are similar to those captured in the GSC program but more details on relatives at risk was collected. |
| **Primary analysis** | To what extent are all data included in the analysis of the primary outcome? | 5 | Any decisions made to the primary analysis during the trial maintained pragmatic nature of study. |

|  | Strategy Selected for At Least One Relative | | | | |  |
| --- | --- | --- | --- | --- | --- | --- |
|  | Family and HCP Packet (N=106) | Cascade Chatbot (N=56) | FH Outreach and Support Program (N=16) | **Any strategy (N=121)** | **None (N=54)** | P-value^1^ |
| **Age at result receipt** |  |  |  |  |  | <0.0001 |
| Median (IQR) | 62.7 (48.3, 71.4) | 57.4 (45.6, 71.0) | 67.4 (53.4, 71.1) | 61.0 (47.6, 71.4) | 50.1 (36.7, 66.0) |  |
| Range | 21.1, 89.0 | 21.1, 89.0 | 27.5, 84.6 | 21.1, 89.0 | 21.7, 80.5 |  |
| **Sex**, n (%) |  |  |  |  |  | 0.5584 |
| Male | 45 (42.5%) | 21 (37.5%) | 7 (43.8%) | 48 (39.7%) | 24 (44.4%) |  |
| Female | 61 (57.5%) | 35 (62.5%) | 9 (56.3%) | 73 (60.3%) | 30 (55.6%) |  |
| **Race**, n (%) |  |  |  |  |  | 0.3089 |
| White | 105 (99.1%) | 54 (96.4%) | 16 (100.0%) | 119 (98.4%) | 51 (94.4%) |  |
| Other^2^ | 1 (0.9%) | 2 (3.6%) | 0 (0.0%) | 2 (1.6%) | 3 (5.6%) |  |
| **Ethnicity**, n (%) |  |  |  |  |  | 0.3666 |
| Non-Hispanic or Latino | 105 (99.1%) | 55 (98.2%) | 16 (100.0%) | 120 (99.2%) | 52 (96.3%) |  |
| Other^3^ | 1 (0.9%) | 1 (1.8%) | 0 (0.0%) | 1 (0.8%) | 2 (3.7%) |  |
| **State**, n (%) |  |  |  |  |  | 0.3089 |
| Not PA | 2 (1.9%) | 1 (1.8%) | 1 (6.3%) | 2 (1.6%) | 3 (5.6%) |  |
| PA | 104 (98.1%) | 55 (98.2%) | 15 (93.8%) | 119 (98.4%) | 51 (94.4%) |  |
| **Insured Type** |  |  |  |  |  | 0.7630 |
| Private only | 43 (41.4%) | 30 (54.6%) | 6 (37.5%) | 53 (43.8%) | 24 (47.1%) |  |
| Medicare/Medicaid combinations | 61 (58.6%) | 25 (45.4%) | 10 (62.5%) | 66 (54.6%) | 27 (52.9%) |  |
| No insurance | 2 | 1 | 0 | 2 | 3 |  |
| **Variant**, n (%) |  |  |  |  |  | 0.0506 |
| APOB | 43 (40.6%) | 23 (41.1%) | 5 (31.3%) | 49 (40.5%) | 14 (25.9%) |  |
| LDLR | 63 (59.4%) | 33 (58.9%) | 11 (68.8%) | 72 (59.5%) | 40 (74.1%) |  |
| **Completed a genetic counseling appointment**, n (%) | 53 (50.0%) | 36 (64.3%) | 13 (81.3%) | 65 (53.7%) | 16 (29.6%) | 0.0033 |
| ^1^Type 3 GEE Wald statistic p-value value comparing Any Strategy Selected vs. None | | | | | | |

**Supplemental Table 2.** Probands Demographic Characteristics by Strategy Selected

**Supplemental Table 3.** All  and First Degree Relatives Demographic Characteristics by Any Strategy Selected

|  | All Relatives | | | First Degree Relatives | | |
| --- | --- | --- | --- | --- | --- | --- |
|  | Any Strategy Selected (N=541) | None (N=1374) | P-value^1^ | Any Strategy Selected (N=378) | None (N=347) | P-value^1^ |
| **Cascade Uptake**, n (%) |  |  | <0.0001 |  |  | <0.0001 |
| Not completed | 397 (73.4%) | 1341 (97.6%) |  | 265 (70.1%) | 321 (92.5%) |  |
| Completed | 144 (26.6%) | 33 (2.4%) |  | 113 (29.9%) | 26 (7.5%) |  |
|  |  |  |  |  |  |  |
| **Cholesterol/Genetic Testing**, n (%) |  |  |  |  |  |  |
| *Cholesterol testing completed* | *75 (52.1%)* | *30 (90.9%)* | *<0.0001* | *65 (57.5%)* | *23 (88.5%)* | *0.0115* |
| Positive/High lipid result | 49 (65.3%) | 23 (76.7%) |  | 42 (64.6%) | 20 (87.0%) |  |
| Negative/Normal lipid result | 26 (34.7%) | 7 (23.3%) |  | 23 (35.4%) | 3 (13.0%) |  |
| *Genetic testing completed* | *47 (32.6%)* | *1 (3.03%)* | *<0.0001* | *29 (25.7%)* | *1 (3.85%)* | *0.0011* |
| Positive genetic result | 16 (34.0%) | 0 (0.0%) |  | 10 (34.5%) | 0 (0.0%) |  |
| Negative genetic result | 29 (61.7%) | 1 (100.0%) |  | 17 (58.6%) | 1 (100.0%) |  |
| Unknown genetic result | 2 (4.3%) | 0 (0.0%) |  | 2 (6.9%) | 0 (0.0%) |  |
| *Both completed* | *22 (15.3%)* | *2 (6.06%)* | *0.0014* | *19 (16.8%)* | *2 (7.69%)* | *0.0380* |
| Positive/High lipid result &  Positive genetic result | 16 (72.7%) | 2 (100.0%) |  | 14 (73.7%) | 2 (100.0%) |  |
| Positive/High lipid result &  Negative genetic result | 2 (9.1%) | 0 (0.0%) |  | 1 (5.3%) | 0 (0.0%) |  |
| Negative/Normal lipid result &  Negative genetic result | 4 (18.2%) | 0 (0.0%) |  | 4 (21.0%) | 0 (0.0%) |  |
|  |  |  |  |  |  |  |
| **Genetic Testing**, n (%) |  |  | <0.0001 |  |  | 0.0004 |
| Not completed/Pending | 472 (87.2%) | 1371 (99.8%) |  | 330 (87.3%) | 344 (99.1%) |  |
| Completed | 69 (12.8%) | 3 (0.2%) |  | 48 (12.7%) | 3 (0.9%) |  |
| Positive genetic result | 32 (46.4%) | 2 (66.7%) |  | 24 (50.0%) | 2 (0.6%) |  |
| Negative genetic result | 35 (50.7%) | 1 (33.3%) |  | 22 (45.8%) | 1 (0.3%) |  |
| Unknown genetic result | 2 (2.9%) | 0 (0.0%) |  | 2 (4.2%) | 0 (0.0%) |  |
|  |  |  |  |  |  |  |
| **Cholesterol Testing**, n (%) |  |  | <0.0001 |  |  | 0.0024 |
| Not completed | 444 (82.1%) | 1342 (97.7%) |  | 294 (77.8%) | 322 (92.8%) |  |
| Completed | 97 (17.9%) | 32 (2.3%) |  | 84 (22.2%) | 25 (7.2%) |  |
| Positive/High lipid result | 67 (69.1%) | 25 (78.1%) |  | 57 (67.9%) | 22 (88.0%) |  |
| Negative/Normal lipid result | 30 (30.9%) | 7 (21.9%) |  | 27 (32.1%) | 3 (12.0%) |  |
|  |  |  |  |  |  |  |
| **Age, years** |  |  | 0.0533 |  |  | 0.0200 |
| Median (IQR) | 53.0 (36.0, 65.5) | 55.0 (35.0, 70.0) |  | 52.0 (37.0, 64.0) | 57.0 (43.0, 70.0) |  |
| Missing | 1007 | 915 |  | 181 | 161 |  |
|  |  |  |  |  |  |  |
| **Sex**, n (%) |  |  | 0.4147 |  |  | 0.7539 |
| Female | 242 (48.3%) | 628 (51.0%) |  | 190 (50.3%) | 168 (51.5%) |  |
| Male | 259(51.7%) | 604 (49.0%) |  | 188 (49.7%) | 158 (48.5%) |  |
| Missing | 40 | 142 |  | 0 | 21 |  |
|  |  |  |  |  |  |  |
| **Race**, n (%) |  |  | >0.9999* |  |  | >0.9999* |
| White | 72 (98.6%) | 33 (100.0%) |  | 61 (98.4%) | 25 (100.0%) |  |
| Other^2^ | 1 (1.4%) | 0 (0.0%) |  | 1 (1.6%) | 0 (0.0%) |  |
| Missing | 468 | 1341 |  | 316 | 322 |  |
|  |  |  |  |  |  |  |
| **Ethnicity**, n (%) |  |  | --- |  |  | --- |
| Non-Hispanic or Latino | 55 (100.0%) | 32 (100.0%) |  | 47 (100.0%) | 24 (100.0%) |  |
| Missing | 486 | 1342 |  | 331 | 323 |  |
|  |  |  |  |  |  |  |
| **State**, n (%) |  |  | 0.3538 |  |  | 0.2085 |
| Not PA | 62 (13.6%) | 16 (10.1%) |  | 50 (14.3%) | 11 (9.1%) |  |
| PA | 393 (86.4%) | 143 (89.9%) |  | 299 (85.7%) | 110 (90.9%) |  |
| Missing | 86 | 1215 |  | 29 | 226 |  |
| *Fisher’s exact test | | | | | | |
